# Substantial Intestinal Microbiota Differences Between Patients With Ulcerative Colitis From Ghana and Denmark

**DOI:** 10.3389/fcimb.2022.832500

**Published:** 2022-03-03

**Authors:** Hengameh Chloé Mirsepasi-Lauridsen, Katleen Vranckx, Henrik Vedel Nielsen, Lee O’Brien Andersen, Timothy Archampong, Karen Angeliki Krogfelt, Andreas Munk Petersen

**Affiliations:** ^1^ Department of Bacteria, Parasites and Fungi, Statens Serum Institut, Copenhagen, Denmark; ^2^ Department of Science and Environment, Unit of Molecular and Medical Biology, Roskilde University, Roskilde, Denmark; ^3^ Applied Maths NV, Sint-Martens-Latem, Belgium; ^4^ Department of Medicine and Therapeutics, University of Ghana Medical School, Korle-Bu, Accra, Ghana; ^5^ Department of Virus and Microbiological Special Diagnostics, Statens Serum Institut, Copenhagen, Denmark; ^6^ Department of Gastroenterology, Copenhagen University Hospital Hvidovre, Hvidovre, Denmark; ^7^ Department of Clinical Microbiology, Copenhagen University Hospital Hvidovre, Hvidovre, Denmark

**Keywords:** ulcerative colitis, prokaryotes/eukaryotes, African diet, Westernized diet, inflammatory bowel disease (IBD), *Escherichia coli*

## Abstract

**Background:**

Ulcerative colitis (UC) is a relapsing nontransmural inflammatory disease that is restricted to the colon and is characterized by flare-ups of bloody diarrhea. In this study, we aimed to investigate intestinal bacterial diversity in healthy controls and patients with UC with and without active disease, from Ghana and Denmark.

**Methods:**

The study included 18 UC patients (9 with active and 9 with inactive disease) and 18 healthy controls from Ghana. In addition 16 UC patients from Denmark (8 UC with active and 8 UC with inactive disease) and 19 healthy controls from Denmark. Microbiota diversity analysis relied on sequencing of ribosomal small subunit genes. Purified genomic DNA was submitted to PCR using a primer set targeting prokaryotes and eukaryotes. The purified DNA was sequenced on the Illumina MiSeq system in a 2 × 250 bp set up (Illumina, San Diego, CA, USA). Blinded analysis of the taxonomy table was performed using BioNumerics-7.5 (Applied Maths NV, Sint-Martens-Latem, Belgium).

**Results:**

When analyzing the taxonomy data for prokaryotes, cluster and principal component analysis shows Danish healthy controls clustered together, but separate from healthy controls from Ghana, which also clustered together. The Shannon diversity index (SDI) for prokaryotes shows significant differences between Danish healthy controls and patients in comparison with the corresponding groups from Ghana (*p* = 0.0056). Significant increased abundance of *Escherichia coli* was detected in healthy controls from Ghana in comparison with healthy controls from Denmark. The SDI of the prokaryotes ranges between 0 and 3.1 in the Ghana study groups, while in the Danish study groups it ranges between 1.4 and 3.2, the difference is however not significant (*p* = 0.138). Our data show a significant increased abundance of eukaryotes species in the healthy control group from Ghana and Denmark in comparison with patient groups from Ghana and Denmark.

**Conclusion:**

Overall, healthy controls and patients with UC from Denmark have increased diversity of prokaryotes. Healthy controls from Denmark and Ghana have increased abundance of eukaryotes in comparison with UC patient groups from Denmark and Ghana.

## Introduction

Ulcerative colitis (UC) is a chronic inflammatory disease of the colon characterized by bloody diarrhea and abdominal pain (Baumgart and Sandborn, 2007). The etiology of UC is unknown, but the findings so far suggest that the nexus of UC pathogenesis lies in the interaction between the predisposing host genetic factors and the host immune response to intestinal bacteria (Baumgart and Sandborn, 2007). As the prevalence of UC increases in Western countries by 2%–3% each year, an increased attention has been given toward the role of intestinal microbiota and diet in UC (Annese et al., 2003; Loftus et al., 2007). Therefore, it is interesting to examine the nature of the intestinal microbiota composition in both active and inactive UC in a Western country such as Denmark and compare it with a non-Western country such as Ghana.

In UC patients it is shown an increased prevalence of Actinobacteria and Proteobacteria such as *Escherichia coli* and decreased prevalence of Clostridial cluster IV such as *Faecalibacterium prausnitzii* (Frank et al., 2007; Sokol et al., 2008) and Firmicutes such as *Lactobacillus* (Frank et al., 2007; Strober, 2013; Vester-Andersen et al., 2019; Nascimento et al., 2020). Actinobacteria phylum are known to have a pivotal role in maintaining gut homeostasis and metabolism (Binda et al., 2018). However, the abundance of *Coriobacteriia* and family *Coriobacteriaceae*, which is known to metabolize host-derived bile acids and steroid hormones, was increased in IBD, while the family *Bifidobacteriaceae* was reduced (Alam et al., 2020). Reduced abundance of *Lactobacillus* species, lactic acid-producing bacteria is linked to UC, and lactic acid-producing bacteria are known to benefit the gut by creating an intestinal environment, not favorable for pathogenic bacteria (Walter, 2008). Studies in UC patients indicate increased abundance of virulent *E. coli* species, harboring virulent genes such as alpha-hemolysin, which causes increased gut permeability (Mirsepasi-Lauridsen et al., 2016; Mirsepasi-Lauridsen et al., 2020). *Faecalibacterium prausnitzii* produces short chain fatty acid (SCFA) from dietary carbohydrate, which provides energy to colonocytes and have anti-inflammatory properties (Frank et al., 2007). Reduced level of SCFA might be explained by the reduced abundance of *Faecalibacterium prausnitzii* in UC patients. An important element in this endeavor is nutrition. Diet intake of fast-food, rich in fats and digestible sugar, increases the risk of UC (Burisch et al., 2014; Nascimento et al., 2020), whereas diet rich in olive oil, fish, fruits, and nondigestible fibers such as vegetables seem to be protective against UC (D’Souza et al., 2008; Ananthakrishnan et al., 2013). Nutrition therapy has been shown to be as effective as corticosteroids for mucosa healing in UC patients (Borrelli et al., 2006).

It has been speculated that there might be a link between environment, diet, microbiota, and the decreased prevalence of inflammatory bowel disease (IBD) in Asia and Africa (Nkrumah, 2008; Isibor et al., 2021). Decreased incidence of UC in Asian and African countries might be due to under reporting and/or limited access to hospitals and healthcare in general.

Increased use of antibiotics early in life has been linked to an increased risk of developing IBD (Jin et al., 2017; Vangoitsenhoven, 2020). However, these results do not match with the increased use of antibiotics without prescription in some Asian and African countries (Dixon et al., 2019) and decreased incidence of IBD in these countries. As mentioned earlier, there are studies investigating intestinal prokaryotes in patients with IBD in comparison with the healthy controls, but only a few studies investigating the prevalence of eukaryotes in IBD in comparison with healthy controls in Western countries versus Africa.

Increased prevalence of parasites such as *Trichuris suis* among Asians and Africans has been linked to more mature immunity and protection against autoimmune diseases such as IBD. IBD patients treated with *Trichuris suis* ova, improved their symptoms and maintained remission (Huang et al., 2018). This might suggest why the Asian and African populations are protected against autoimmune diseases such as IBD.

This study aimed to investigate the intestinal bacteria and eukaryote diversity in healthy controls and in UC patients with active and inactive disease from Ghana and Denmark.

## Materials and Methods

### Study Population

This is a descriptive study of intestinal microbiota in UC patients with active and inactive disease from Denmark and Ghana. The study includes 17 adult patients with active ulcerative colitis (UC) disease (9 from Ghana, 8 from Denmark) and 17 UC patients with inactive disease (9 from Ghana, 8 from Denmark). In addition, the study includes 37 healthy controls (18 healthy persons from Ghana and 19 from Denmark). The age of UC patients from Ghana ranges from 20 to 70 years, while the ages of the UC patients from Denmark range from 23 to 64 years. The age of healthy controls from Ghana ranges from 23 to 72, while the age of the healthy controls from Denmark ranges from 20 to 70 years.

### Clinical Assessment of Study Population

UC patients were diagnosed according to standardized criteria (Walmsley et al., 1998) with symptoms as bloody diarrhea, tenesmus, and rectal urgency. Patients with severe disease will in up to 47% of the cases have extraintestinal manifestations, such as uveitis, arthritis, erytema nodosum (Langan et al., 2007). Patients suspected of UC should be tested for *Clostridium difficile* infection/toxin, parasitic ova, and parasites, as well as pathogenic *E. coli*, to eliminate other causes of chronic diarrhea. The diagnosis of UC is supported by inflammation markers in blood test, fecal-calprotectin test, colonoscopy, proctosigmoidoscopy, and biopsy (Langan et al., 2007). Symptom scores and fecal and blood samples were collected. The disease activity was assessed by the Simple Clinical Colitis Activity Index (SCCAI) (Ring et al., 2017). SCCAI is a symptom scoring questionnaire regarding day/night bowel frequency, urgency of defecation, blood in feces, general well-being, and extraintestinal manifestations. Scoring ranges between 0 and 19. A SCCAI score of ≤2 was defined as remission, 3–5 as mild disease activity, 6–11 as moderately active disease, and >12 as severely active disease. Patients from Ghana with active disease have pancolitis with SCCAI score of >6. Patients from Denmark with active disease have left-sided colitis with SCCAI sore of >3. Inactive disease was monitored similarly by SCCAI questionnaire, inflammation-marker blood tests (CRP), and fecal calprotectin.

All the participants filled in questionnaires regarding medicine or antibiotic usage and travel activity during the last 3 months before participating in the study. Healthy controls who used antibiotics were excluded from the study (**Table 1**). The laboratory staff was blinded to patient data.

**Table 1:** Number of the participants in the study groups, clinical data, medicine and antibiotic use.

Project group, n	Diagnosis	Antibiotic use	Medication at sampling	Site of inflammation at endoscopy
**Ghana**	** **	** **	** **	** **
9	UCA	One particieant used ciprofloxacin	Azathioprine; Prednisolone, Mesalazine; Sulfasalazine; IV Hydrocortisone, Folic acid, Omeprazole	Pancolitis
9	UCI	None	Sulfasalazine; Folic acid; Ferrous sulfate, Mesalazine; Prednisolone; Omeprazole, Pentasa enema	pancolitis
18	Healthy Controls	None	None	None
**Denmark**	** **	** **	** **	** **
8	UCA	None	Prednisolone, Imurel, Asacol, Seretide, Puri-nethol	Left-sided colitis
8	UCI	None	Asacol, Mesasal supp, Pentasa	Left-sided colitis
19	Healthy Controls	None	None	None

### Ethical Statement

Ethics statement permission for the study was obtained from the Regional Ethics Committee for Copenhagen County Hospitals and Ghana (Permission no. KA-03019, Permission no. KA-20060159), and all participants gave their informed written consent. Healthy controls were recruited among volunteer students. Patients and healthy controls completed a questionnaire about their condition and their medication.

### DNA Extraction From Fecal Samples

Stools of the project participants from Ghana were transported under cold conditions within 1 day by air to the Statens Serum Institut (microbiology laboratory), Denmark. Danish project participants send their stool to the Statens Serum Institut (microbiology laboratory) *via* fast mail (same-day delivery). All the stools were stored at −80° degrees until use. DNA extraction of all project participants’ stools was performed according to the instructions of the manufacturer (DNA Stool Mini Kit, Qiagen, Copenhagen, Denmark) with the following modifications: 100 mg fecal sample was mixed with 1.4 ml ASL buffer in a 2-ml tube and vortexed until the sample was thoroughly homogenized. Samples were subsequently mixed with 0.2 g sterile zirconia/silica beads. Hereafter, the samples were processed on a TissueLyser for 6 min at 30 Hz. Lysis was completed at a temperature of 95°C for 5 min. Finally, DNA were eluted in 100 µl elution buffer provided in the kit.

### Sequencing of Microbial Population in Fecal Samples

Microbiota diversity analysis relied on sequencing of the ribosomal small subunit (SSU rRNA) genes. Purified genomic DNA was submitted to PCR using a primer set targeting prokaryotes and eukaryotes [one primer set for 16S, and three different in-house primer sets for 18S (G3-1, G4-3, G6-1)]. For prokaryotes, a modified version of the published universal prokaryotic primers 341F/806R (Yu et al., 2005) was used. Resulting PCR products were quantified using the Quant-IT™ dsDNA High Sensitive Assay Kit (Thermo Fisher Scientific, Waltham, MA, USA) and pooled in equimolar amounts [Pooled Amplicon Library (PAL)]. Agencourt AMPure XP Beads (Beckman Coulter, Brea, CA, USA) were used to remove DNA fragments shorter than 300 bp and those longer than 1,000 bp, and the purified DNA was sequenced on the Illumina MiSeq system in a 2 × 250-bp set up (Illumina Inc.). A maximum of 64 samples were sequenced in a single sequencing run (Ring et al., 2017). The sequence output was taxonomically mapped using BION, a newly developed k-mer-based mapping software. A k-mer length of 8 was used, with a step size of 4. Query sequences originating from prokaryotes were compared with the 341–806 bp region (rRNA gene positions from *Escherichia coli*) in RDP 11.04 (30).

### Data Analysis

Blinded analysis of the taxonomy table was performed using BioNumerics version 7.5 (Applied Maths NV, Sint-Martens-Latem, Belgium). After normalization, cluster analysis was performed with a tolerance of 1% and an optimization of 1%. The similarity between profiles was calculated with a Pearson’s correlation. A dendrogram was then constructed with UPGMA. The reliability of the dendrogram was determined with a cophenetic correlation coefficient. This coefficient compares a similarity matrix derived from the dendrogram with the actual similarity matrix. Reliably separated branches have a high cophenetic correlation. The Shannon Diversity Index was calculated for each profile. Linear discriminant analysis (LDA) was used to analyze differences within patient groups and healthy persons. All statistical analyses were performed after mean-based normalization using analysis of variance (ANOVA) with Bonferroni *post-hoc* test and paired and unpaired *t*-tests. The level of significance was set at *p* ≤ 0.05.

## Results

**Figure 1:**
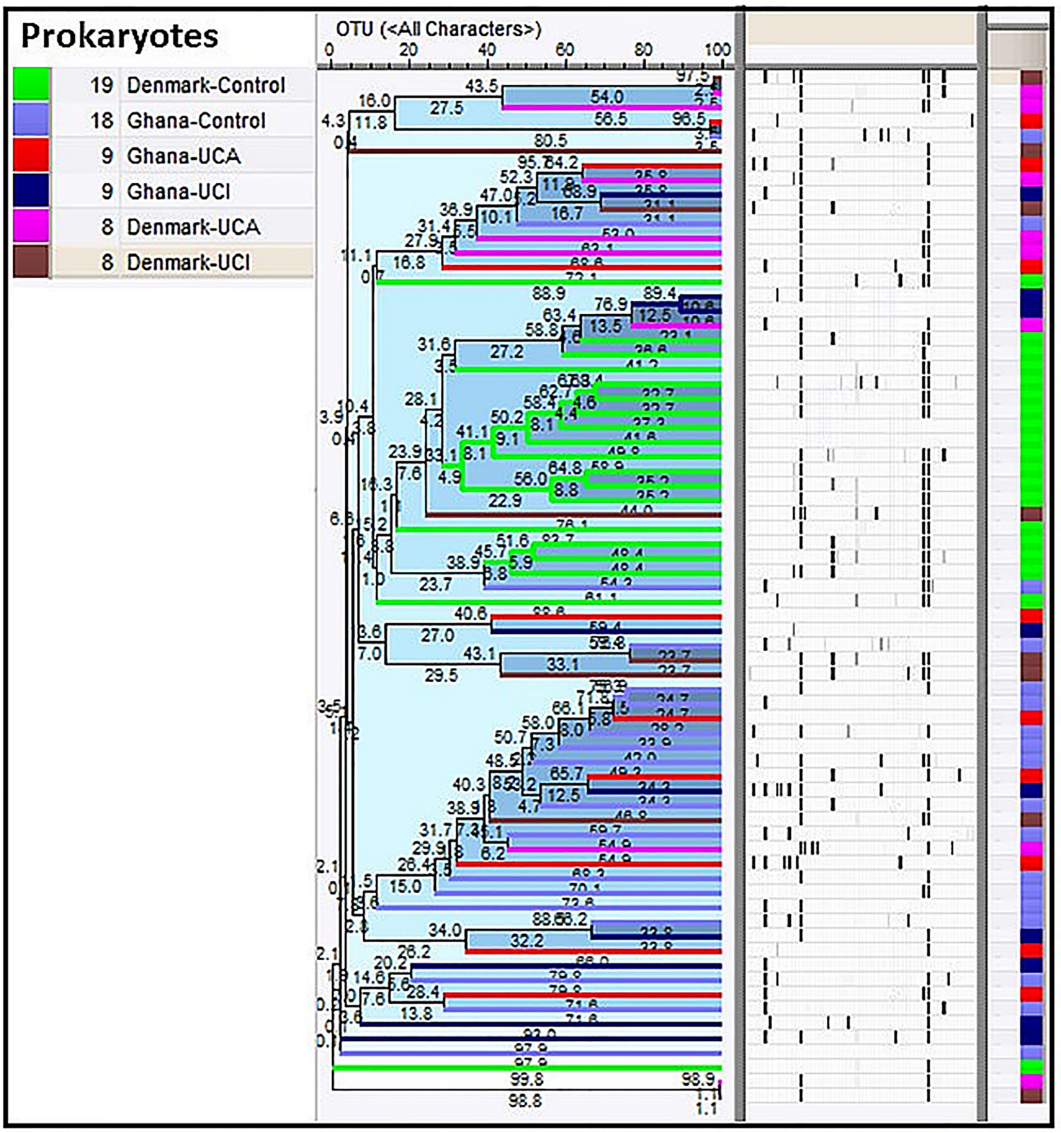
Cluster analysis of prokaryotes in study groups show healthy controls from Denmark are clustered together in the middle of the dendrogram (in green), while 16 of the healthy controls from Ghana are clustered together with patients from Ghana in the bottom of the dendrogram.

Cluster analysis shows that 18 of 19 healthy controls from Denmark are mostly clustered together in the middle of the dendrogram, while 16 of 18 healthy controls from Ghana are clustered together with patients from Ghana in the bottom of the dendrogram (**Figure 1**).

**Figure 2:**
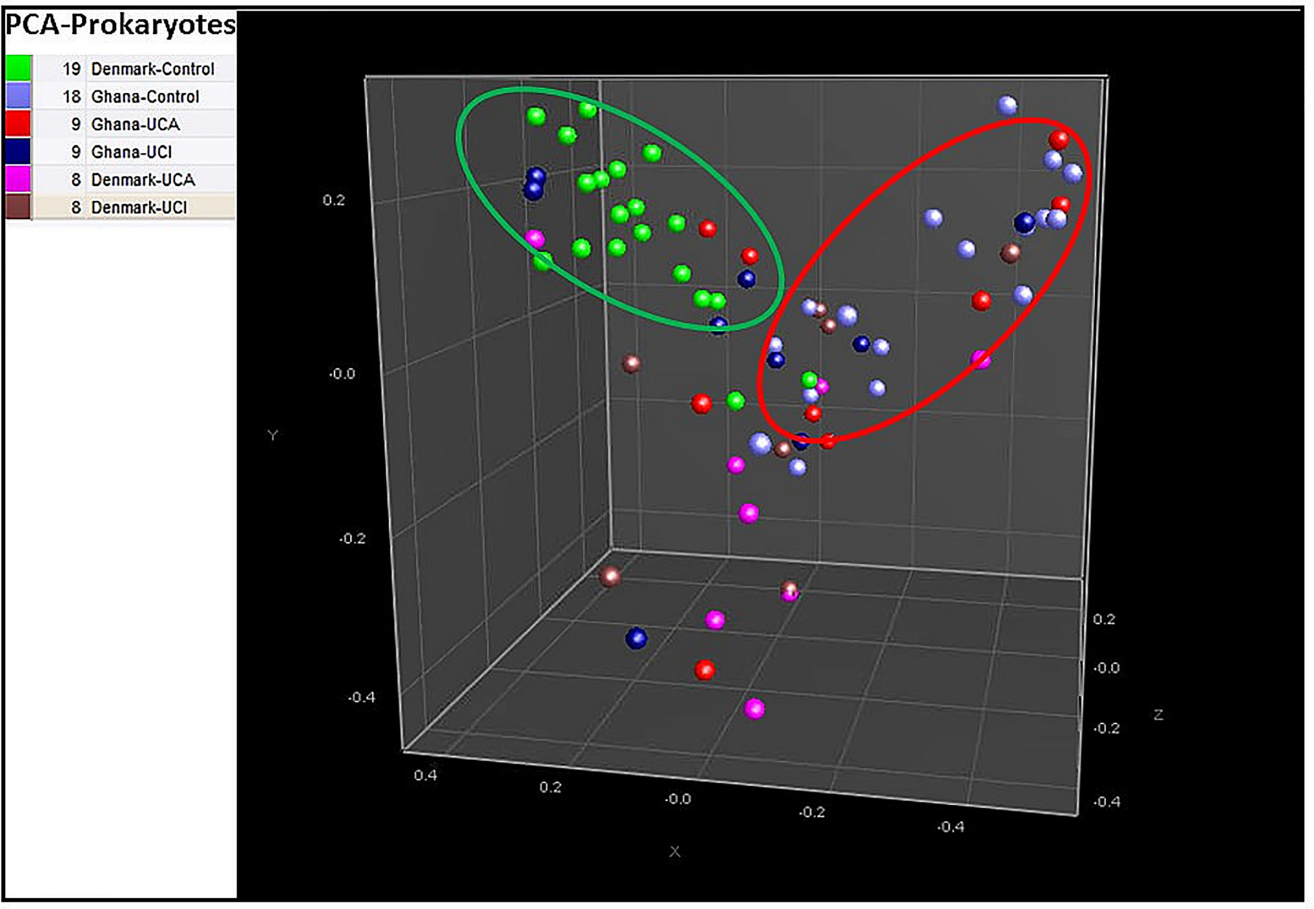
Principal component analysis (PCA) based on prokaryotic taxonomy results show 94% of healthy controls from Denmark are in the middle of the PCA in green circle, while 88% of the healthy controls from Ghana are clustered together with UC patients from Denmark and Ghana in the red circle.

Principal component analysis (PCA) based on the prokaryotic taxonomy results shows that 94% of the Danish healthy controls are mostly clustered together in the green circle in **Figure 2**, while 88% of healthy controls from Ghana are mostly clustered together with UC patients from Denmark and Ghana in the red circle (**Figure 2**).

**Figure 3:**
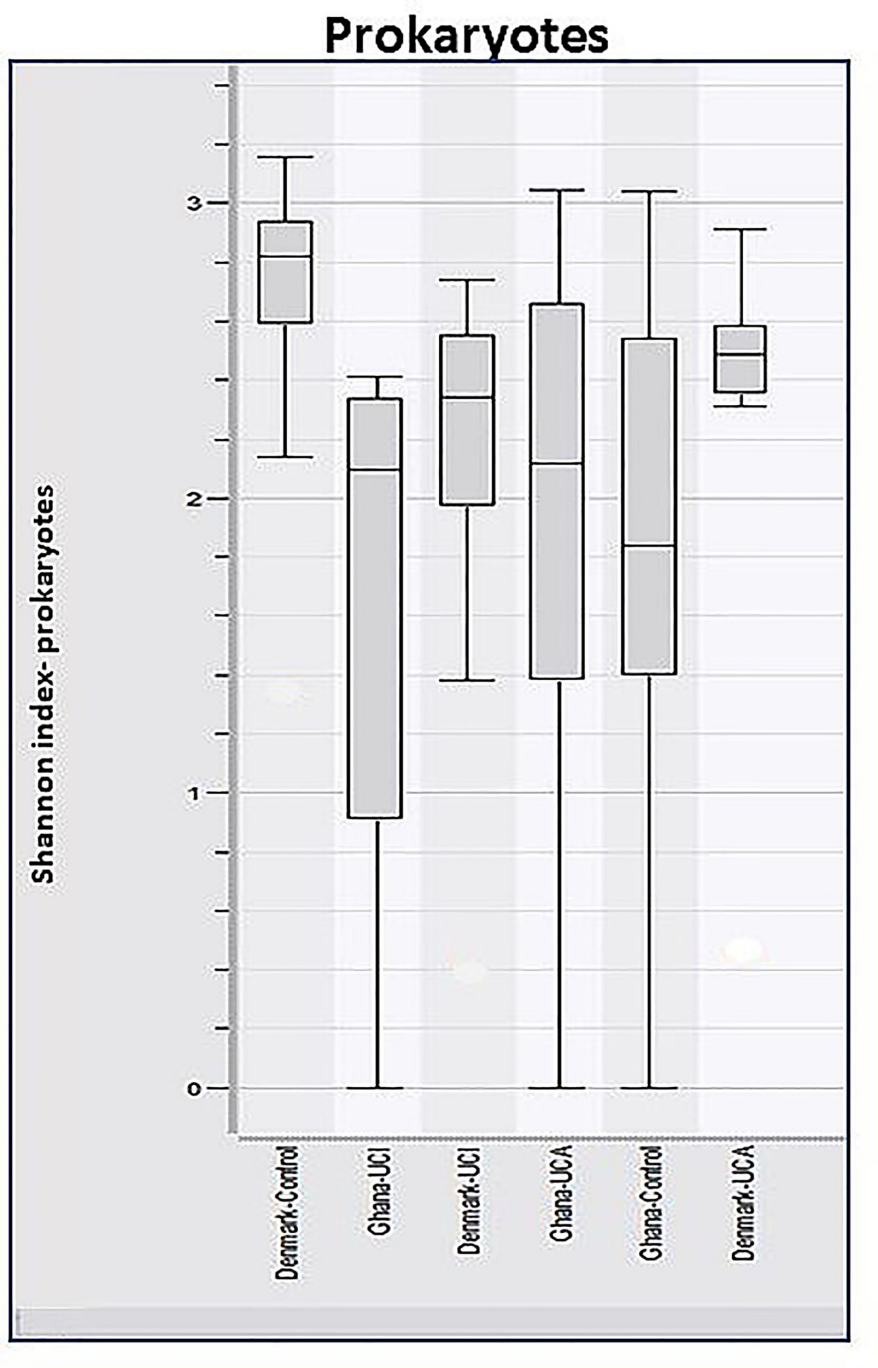
Boxplots of calculated Shannon Diversity Index, divided by study groups show increased prokaryotic diversity in Danish healthy controls and patient groups, in comparison with healthy control and patient group from Ghana, *p* ≈ 0.0056.

Analysis of α-diversity using the Shannon Diversity Index, and two-way-ANOVA test on ranks showed significant differences between Danish healthy controls and patients compared with the healthy controls and patients from Ghana, *p* ≈ 0.0056 (**Figure 3**). The Shannon Diversity Index in healthy controls and patients from Ghana ranges between 0 and 3.1, while the Shannon Diversity Index in the Danish healthy controls and patients ranges between 1.4 and 3.2.

**Figure 4:**
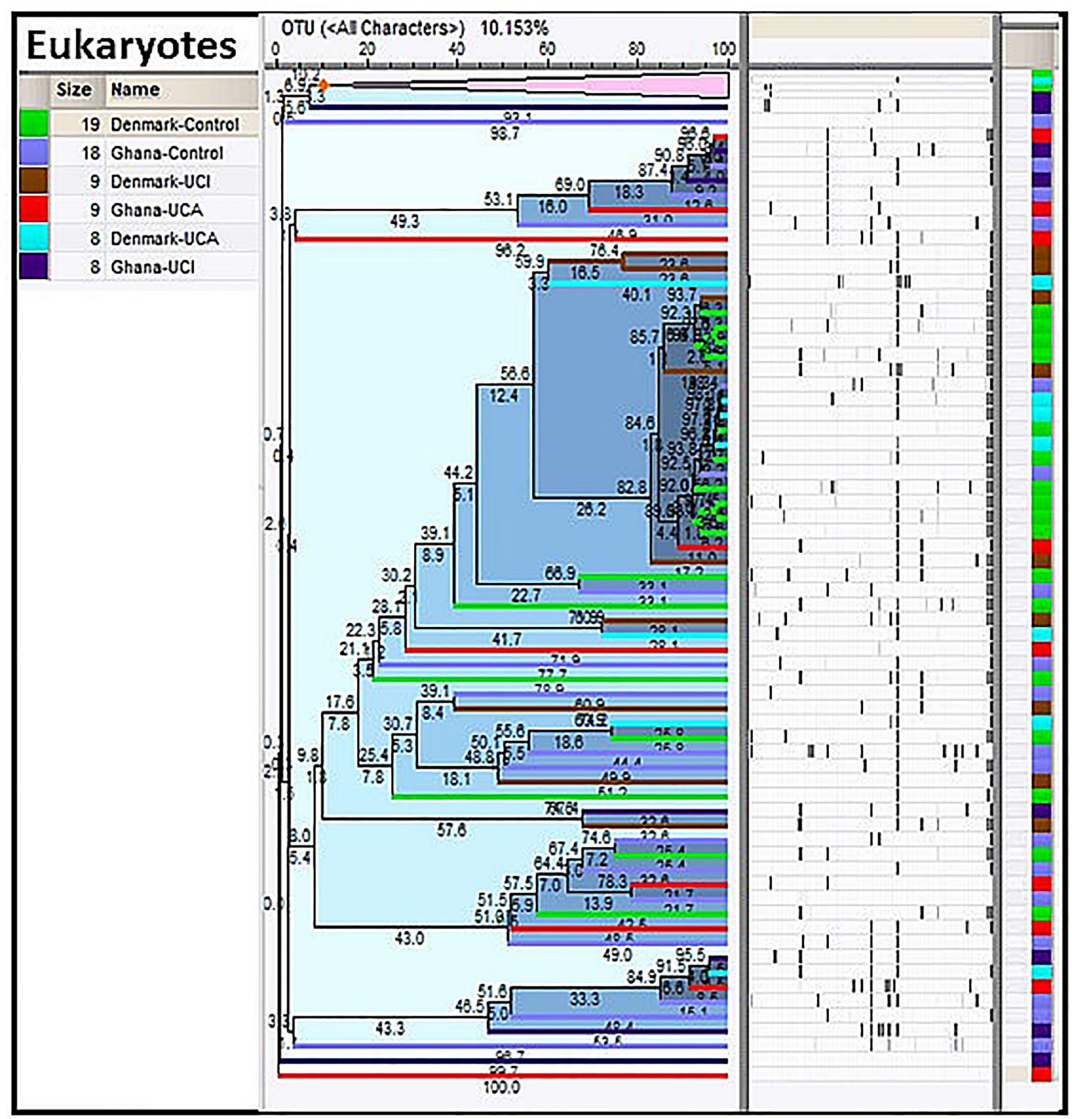
Cluster analysis of eukaryotes in the study population. As it is shown, the Danish control group are clustered together in the center of the dendrogram with Danish UC patients, while the study groups from Ghana are spread all over the dendrogram.

Taxonomy data for eukaryotes have been screened for plants, animals, or any other species that are only connected to the human host, through the food chain. Cluster analysis of the taxonomy data for eukaryotes shows the Ghana groups are spread all over the dendrogram, while the Danish groups are mostly gathered in the center of the dendrogram with Danish UCI and UCA (**Figure 4**).

**Figure 5:**
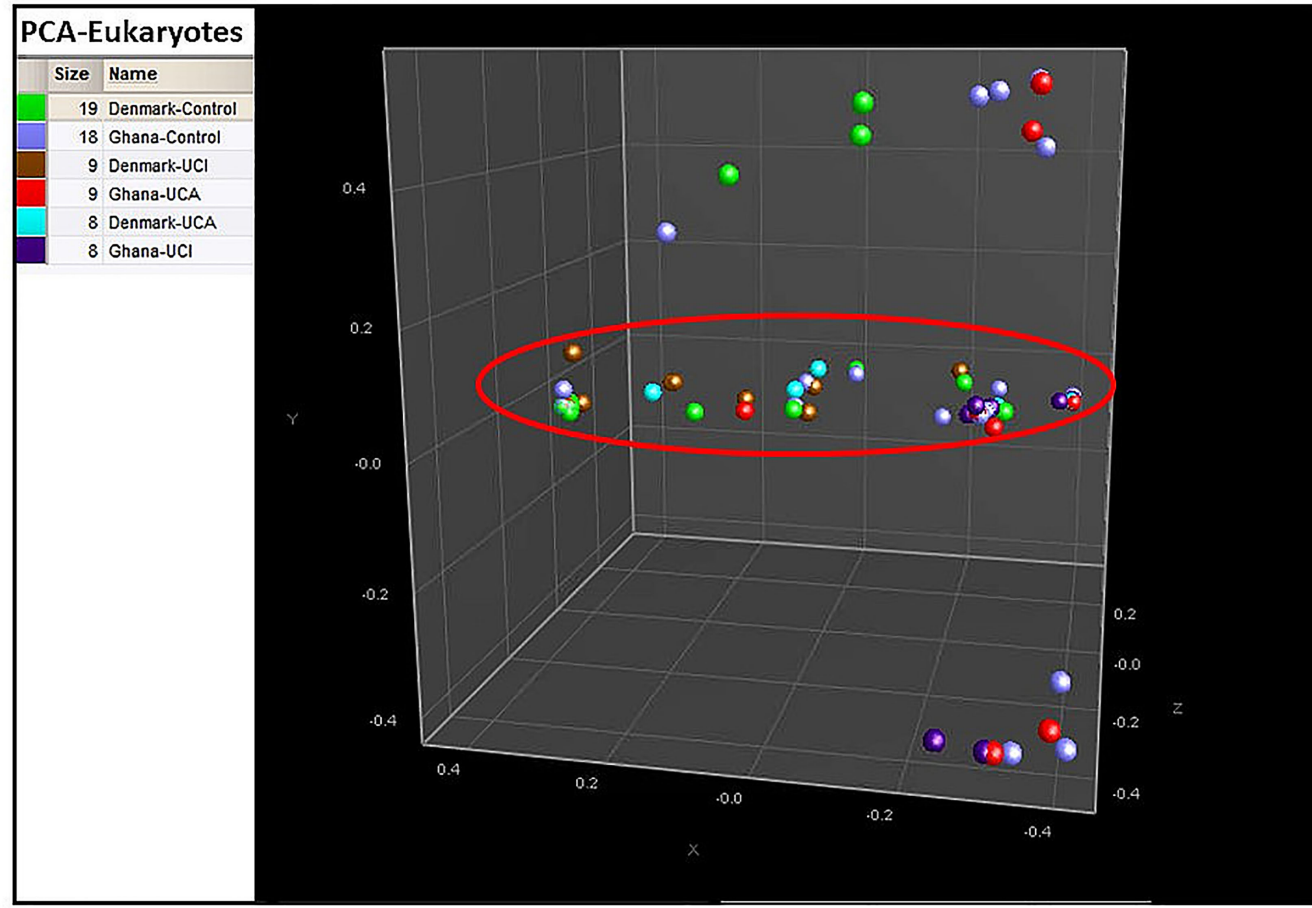
PCA is based on the cluster analysis; 85% of the Danish healthy controls are gathered in the red circle, while healthy controls from Ghana and patient groups are spread all over the PCA.

PCA shows that 85% of the Danish healthy controls are clustered together with 67% of the Danish UCI and 78% of the UCA patients in the red circle, while healthy controls and UCA and UCI patients from Ghana are spread all over the PCA (**Figure 5**).

**Figure 6:**
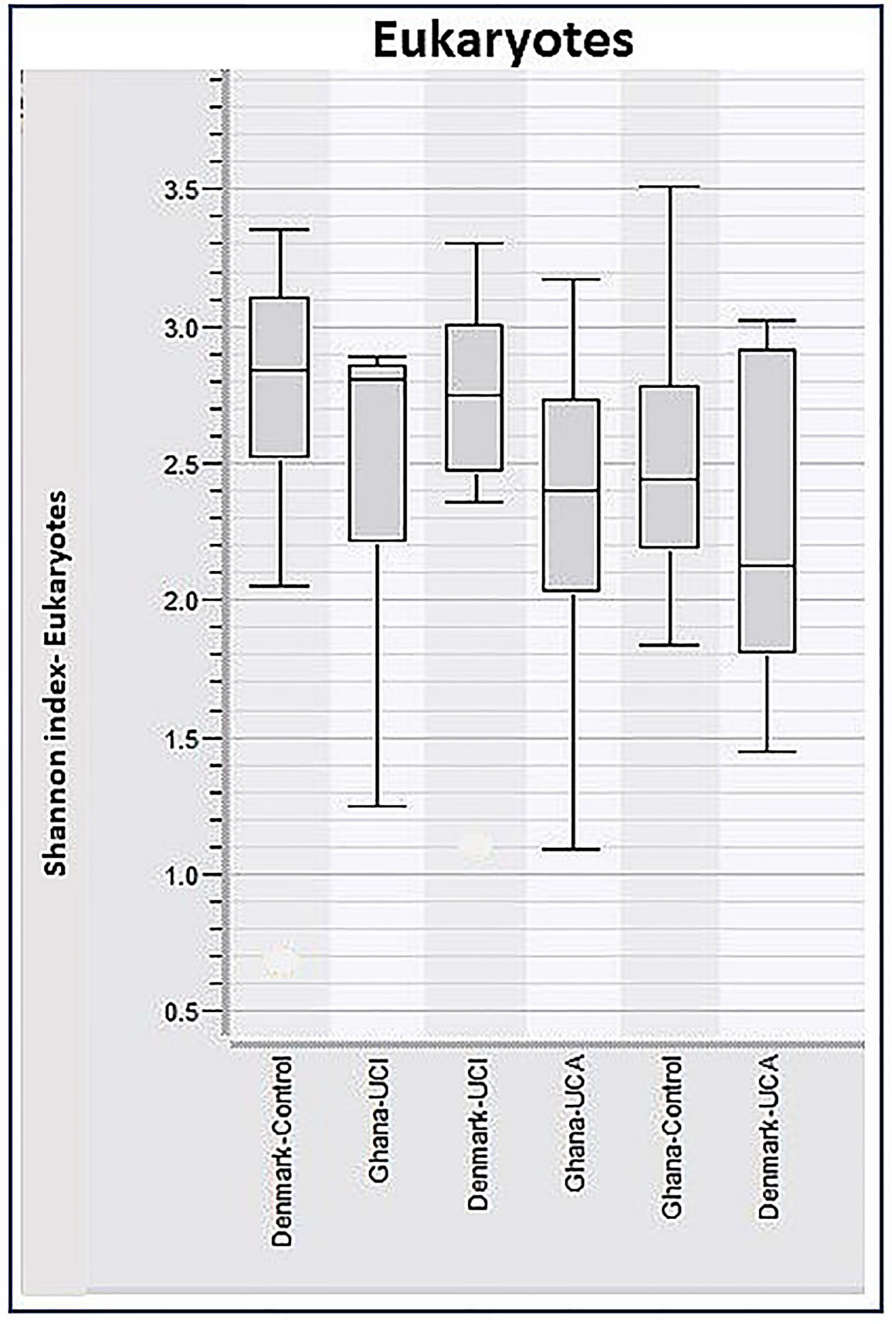
Boxplots of calculated Shannon Diversity Index, divided by groups show increased eukaryotic diversity in healthy controls. However, these differences are not significant.

When analyzing the taxonomy data of eukaryotes for the Shannon Diversity Index, a two-way ANOVA test on ranks shows no significant differences between the Danish and Ghana study groups, *p* = 0.401 (**Figure 6**).

**Figure 7:**
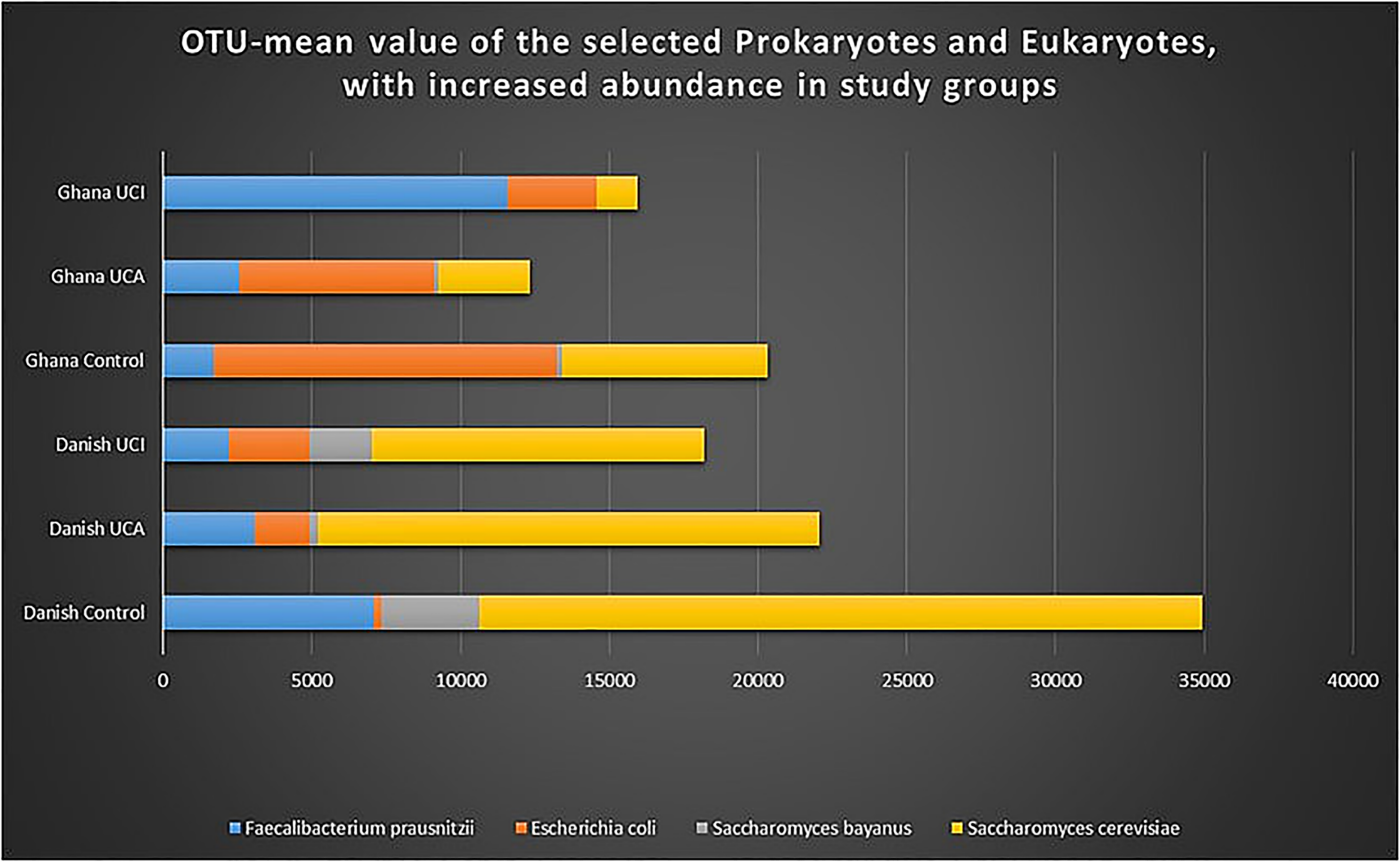
Graph shows OTU-mean value for selected prokaryotes and eukaryotes with increased abundance. As it is shown in the graph, *E. coli*, *Saccharomyes*, and *Faecalibacterium* are selected for highest peaks among other species found in the stool of the study groups.

Analysis of operational taxonomic unit (OTU) for prokaryotes shows a significant increased abundance of *E. coli* in the Ghana control group in comparison with the Danish control group, *p* = 0.0001 (**Figure 7**). A significant increase in the abundance of *Faecalibacterium* is noticed in the Danish healthy controls vs. Ghana healthy controls (*p* = 0.0002).

When analyzing OTUs for eukaryotes, a significant increase in the abundance of *Saccharomyces cerevisiae* was detected in the Danish control groups in comparison with Ghana control group (*p* = 0.004) and in the Danish UC patients with inactive disease in comparison with UC patient with inactive disease from Ghana (*p* = 0.05). A significant increase in the abundance of *Saccharomyces bayanus* was also detected in the Danish control groups in comparison with the Ghana control group (*p* = 0.01) and in the Danish UC patients with inactive disease in comparison with UC patient with inactive disease from Ghana (*p* = 0.05).

The data show that the healthy control groups from Denmark and Ghana have a significant increase in the abundance of eukaryotes compared with the UC patient groups from Denmark and Ghana, *p* = 0.0001. When analyzing each group independently, it is shown that individual healthy control participants from Denmark and Ghana have increased abundance of *Amoebozoa*, *Ascomycota*, *Basidiomycota*, and *Stramenopiles*; the median value is, however, zero for all study groups (**Table 2**).

**Table 2:** Median value for selected Eukaryota in study groups.

Study groups	*Amoebozoa*		*Ascomycota*		*Basidiomycota*		Stramenopiles Blastocystida	
	Median	Total	Median	Total	Median	Total	Median	Total
Danish healthy control	0	7	0	681,367	0	13,631	0	181,821
Danish UCI	0	48	0	153,729	0	6,253	0	7,145
Danish UCA	0	0	0	273,470	0	13,385	0	0
Ghana healthy control	0	24,671	0	353,251	0	12,938	0	103,987
Ghana UCI	0	25,236	0	191,078	0	20,480	0	0
Ghana UCA	0	0	0	306,847	0	3,924	0	80,605

When comparing the study groups, the healthy control groups from Denmark and Ghana have increased prevalence of *Amoebozoa*, *Ascomycota*, *Basidiomycota*, and *Stramenopiles*; however, these differences are not statistically significant (**Table 3**).

**Table 3:** P-value for t-test for significant differences between Eukaryota abundance of the 2 selected study groups.

Study Groups	Ascomycota	Amoebozoa	Stramenopiles Blastocystida	Basidiomycota
Danish Healthy Control VS Danish UCA	0.14	0.20	0.20	0.98
Danish Healthy Control VS Danish UCI	0.12	0.24	0.22	0.26
Ghana-Healthy Control VS Ghana-UCA	0.35	0.06	0.84	0.05
Ghana-Healthy Control VS Ghana UCI	0.09	0.49	0.36	0.24

Overall, the healthy control groups from Denmark and Ghana have a significant increase in the abundance of eukaryotes species in comparison with the UC patient groups from Ghana and Denmark, *p* = 0.0001 (**Table 4**).

**Table 4:** Total OTUs of selected eukaryotic species with increased abundance in the study group.

Genus	Species	Denmark control	Denmark UCA	Denmark UCI	Ghana control	Ghana UCA	Ghana UCI
*Cladosporium*	*cladosporioides*	3,437	105	0	1,694	963	6,561
*Cladosporium*	*herbarum*	2,611	0	903	847	452	3,085
*Aspergillus*	*fumigatus*	1,973	1,904	0	204	3,394	0
*Aspergillus*	*proliferans*	481	0	0	346	84	0
*Aspergillus*	*sclerotiorum*	3,955	0	1,601	0	0	0
*Lacazia*	*loboi*	962	24	0	10,542	0	0
*Hymenoscyphus*	*kiko*	2,307	0	0	0	0	1,723
*Geotrichum*	*candidum*	10,658	202	31,252	4	530	51,730
*Candida*	*albicans*	1,618	0	0	67,188	108,805	43,126
*Candida*	*krusei*	0	73,583	571	88,278	68,253	32,707
*Candida*	*rugosa*	0	3	0	12,238	427	1,327
*Candida*	*tropicalis*	14,383	23,811	12,610	61,947	4,054	2,959
*Debaryomyces*	*hansenii*	10,450	0	5,022	1,167	1,746	0
*Saccharomyces*	*bayanus*	71,222	2,422	12,033	3,452	1,466	0
*Saccharomyces*	*cerevisiae*	516,608	148,111	67,922	73,836	27,144	11,083
*Hanseniaspora*	*uvarum*	159	5,519	9,979	641	0	0
*Fusarium*	*oxysporum*	1,130	453	886	7	0	0
*Fusarium*	*solani*	1,581	4,657	1,176	16	0	0
*Trichocladium*	*asperum*	95	369	2,953	1,830	0	857
*Entyloma*	*ficariae*	688	0	0	0	253	363
*Malassezia*	*equina*	33	0	963	0	355	0
*Malassezia*	*globosa*	4,707	10,586	2,982	320	371	1,229
*Malassezia*	*pubis*	462	1,075	482	605	468	1,263
*Malassezia*	*slooffiae*	1,386	318	69	499	133	94
*Malassezia*	*sympodialis*	374	15	187	67	44	46
*Rhodotorula*	*graminis*	0	20	0	716	508	156
*Sporobolomyces*	*roseus*	0	0	0	458	0	690
*Blastocystis*	*blastocystis*	140,584	0	7,145	101,923	66,624	0
*Blastocystis*	ST1	41,237	0	0	2,064	13,981	0

## Discussion

The pathogenic mechanisms of IBD have been studied intensely, pointing out the importance of the genetic and environmental factors in the development of IBD (Wallace et al., 2014).

In the last two decades, the focus has been on reduced diversity of the intestinal microbiota in IBD patients from Western countries, and this reduced microbiota diversity is thought to play an important role in disease relapses and remission or even in developing IBD (Frank et al., 2007; Vester-Andersen et al., 2019; Zakerska-Banaszak et al., 2021). Food and water are important sources when shaping the intestinal microbiota, as the diet influence the composition of the intestinal microbiota also early in life (Marino, 2007). While Western diet has been linked to increased prevalence of IBD, African diets that are rich in fibers with less fat/red meat being linked to the reduced prevalence of IBD.

Therefore, our results were found to be of major importance in discovering the differences in the gut microbiota in healthy controls and UC patients with active and inactive disease from Ghana in comparison with corresponding groups from Denmark. The Shannon Diversity Index shows a significant increase in intestinal bacterial diversity in the Danish study groups in comparison with the corresponding groups from Ghana. Our previous study based on intestinal bacterial diversity in UC patients with active and inactive disease showed similar patterns, where patients with active disease had increased Shannon Diversity Index in comparison with UC patients with inactive disease, while the healthy control group had increased Shannon Diversity Index correspond to UC patients (Mirsepasi-lauridsen et al., 2018).

However, when analyzing bacteria Shannon Diversity Index, we noticed it ranges between 0 and 3.1 within the participants from Ghana compared with the corresponding participant from Denmark, which ranges from 1.4 to 3.2. This result might be explained by the fact that the Western diet is more uniform and processed in comparison with diets from the corresponding study groups from Ghana. Furthermore, the usage of antibiotics is restricted in Denmark (only by medical prescriptions), which causes limited/uniform exposition to the antibiotic, while this is not the case in Ghana (Danish Health and Medicines Authority, 2013; Yevutsey et al., 2017). These facts might have a major impact on the composition of the intestinal microbiota.

Analysis of the prokaryotic taxonomy shows significantly increased abundance of *E. coli* among Ghana healthy controls in comparison with the Danish healthy controls. It was surprising to find an increased abundance of *E. coli* among the healthy control group from Ghana in comparison with the UC patients with active and inactive disease from Ghana, who had 18% and 31% less abundance of *E. coli*, respectively. The Ghana healthy control groups have a significant increase in abundance of *E. coli* in comparison with the Danish UC patient with active disease (*p* = 0.014). It is important to point out that the increased prevalence of *E. coli* is linked to UC, which is also shown in the graph (**Figure 7**) (Kotlowski et al., 2007; Petersen et al., 2009; Mirsepasi-Lauridsen et al., 2016). The question remains why do we observe the opposite pattern in the study group from Ghana, where there is an increased abundance of *E. coli* among the healthy control group in comparison with the UC patients from Ghana. So far, the studies indicate that *E. coli* is one of the most competitive intestinal bacteria with increased antibiotic resistance properties (Kibret and Abera, 2011). It is also known that it is possible to purchase antibiotics in Ghana without medical prescriptions. This might indicate an increased exposition to antibiotics in Ghana, causing increased prevalence of competitive *E. coli* species among other intestinal bacteria. The explanation for reduced abundance of *E. coli* among the Ghana UC patient groups in comparison with the healthy control group might be explained by the increased use of antibiotics, as antibiotics are widely used, especially when patients suffer from intestinal disorders/diseases such as IBD (Yevutsey et al., 2017). However, the subtype of *E. coli* and the possible number of *E. coli* virulence genes in an individual patient is unknown. Certain *E. coli*, such as *E. coli* Nissle has been suggested to have probiotic abilities, if this is the case among patients from Ghana is unknown (Kruis et al., 2004).

Our data show a significant increase in abundance of *Faecalibacterium prausnitzii* among the Danish healthy controls in comparison with the Ghana healthy controls. However, the study group from Ghana shows the opposite pattern, where there is a high abundance of *Faecalibacterium prausnitzii* among the UC patients compared with the healthy control groups from Ghana (**Figure 7**). So far, studies indicate that *Faecalibacterium prausnitzii* exhibit anti-inflammatory properties and has decreased among IBD patients (Frank et al., 2007; Sokol et al., 2008). However, the current study shows a more complex patten. The prokaryotic taxonomy results from this study confirms the study by Hansen et al. (2019), which shows an increased prokaryotic similarity and diversity within nonindustrialized populations from Tanzania and Botswana. There are other things beside diet that might affect the intestinal bacterial diversity, such as depression, which we have not yet been analyzed in this study (Chen et al., 2021). When it comes to prokaryotic taxonomy for UC patients with active and inactive disease in comparison with healthy controls, the results show no significant differences between UC patients with active and inactive disease or between healthy controls and UC patients in the Danish and Ghana study groups. However, the Danish healthy control group (mean-OTU 2,694) has increased intestinal bacterial diversity in comparison with the UC patients with active (mean-OTU 2,281) and inactive (mean-OTU 2,063) disease. Our findings could provide a significance when considering to treat UC patients with probiotics or fecal microbiota transplantation to change the microbiome associated with UC. The taxonomy data of eukaryotes show a significant increase in abundance of *Saccharomyces bayanus* and *Saccharomyces cerevisiae* among the Danish study groups in comparison with the Ghana study groups. Several studies indicate an abnormal reaction to *Saccharomyces* species among IBD patients compared with healthy controls, as IBD patients respond differently to self and non-self-strains (Schaffer et al., 2007; Di Paola et al., 2020). *Saccharomyces cerevisiae* is known as opportunistic pathogen and used in home-brewed beer and in dietary products (Pérez-Torrado and Querol, 2016). The increased prevalence of the *Saccharomyces* species among the Danish study groups might be explained by an increased consumption of wine, beer, and bread, since *Saccharomyces* species are used in bread, beer, and wine production (Lodolo et al., 2008).

Eukaryote data show a significant increase in abundance of eukaryotic species in healthy controls from Denmark and Ghana, in comparison with UC patient groups from Denmark and Ghana. Healthy control participants from Denmark and Ghana have increased prevalence of *Ascomycota*, *Basidiomycota*, and *Stramenopiles*. *Blastocystis* is one of the most common human nonfungal eukaryotic enter-parasitic organisms in developing countries (Petersen et al., 2013). However, the role of the *Blastocystis* in disease and health is still unknown. Some studies link prevalence of *Blastocystis* to irritable bowel syndrome (diarrhea), while other study link high prevalence of the *Blastocystis* with healthy gut (Petersen et al., 2013; Krogsgaard et al., 2018; Kesuma et al., 2019). Our data confirm the results from Petersen et al. (2013), with a significant increase of *Blastocystis* ST1 in the healthy control groups in comparison with the UC patients.

This study clarified that the decreased incidence of UC in people from Ghana is not linked to increased intestinal bacterial diversity nor increased prevalence of eukaryotic species in the intestine. However, this study is based on a limited number of participants. More studies are needed to clarify the role of intestinal microbiota and UC incidence in Ghana. It might be a combination of the bacteria or eukaryotes causing UC or a single organism that we still do not know of.

## Conclusion

Overall, healthy controls and patients with UC from Denmark have increased diversity of prokaryotes compared to Ghana. Healthy controls from Denmark and Ghana have increased abundance of eukaryotes in comparison with UC patient groups from Denmark and Ghana.

## Data Availability Statement

The data presented in the study are deposited in the European Nucleotide Archive repository, accession number PRJEB49838 (ERP134361). Please find the data in the link bellow: https://www.ebi.ac.uk/ena/browser/view/PRJEB49838?show=reads.

## Ethics Statement

The studies involving human participants were reviewed and approved by the Copenhagen County Hospitals (Permission no. KA-03019, Permission no. KA-20060159). The patients/participants provided their written informed consent to participate in this study.

## Author Contributions

AP, KK, HN, TA, and HM-L: design of the study. HM-L, LA, and KV: formal analysis of the data and experimental setting. HM-L: investigation and writing—original draft preparation. AP, KK, TA, LA, KV, and HM-L: reviewing and editing of the manuscript. AP, KK, HN, TA, and HM-L: supervision and administration of the project. All authors have read and agreed to the published version of the manuscript.

## Funding

The authors would like to thank the Torben and Alice Frimodts foundation for the funding provided to HM-L.

## Conflict of Interest

The authors declare that the research was conducted in the absence of any commercial or financial relationships that could be construed as a potential conflict of interest.

## Publisher’s Note

All claims expressed in this article are solely those of the authors and do not necessarily represent those of their affiliated organizations, or those of the publisher, the editors and the reviewers. Any product that may be evaluated in this article, or claim that may be made by its manufacturer, is not guaranteed or endorsed by the publisher.
